# Diagnostic Value of ^18^F-FDG-PET/CT in Patients with FUO

**DOI:** 10.3390/jcm9072112

**Published:** 2020-07-04

**Authors:** Stamata Georga, Paraskevi Exadaktylou, Ioannis Petrou, Dimitrios Katsampoukas, Vasilios Mpalaris, Efstratios-Iordanis Moralidis, Kostoula Arvaniti, Christos Papastergiou, Georgios Arsos

**Affiliations:** 1 3rd Department of Nuclear Medicine, Aristotle University of Thessaloniki Medical School, Papageorgiou General Hospital, 56403 Thessaloniki, Greece; voulaexadaktylou@hotmail.com (P.E.); giannispetrou@hotmail.com (I.P.); katsampoukas@hotmail.com (D.K.); vmpalaris@yahoo.gr (V.M.); emoral@auth.gr (E.-I.M.); garsos@auth.gr (G.A.); 2 ICU and Antimicrobial Stewardship Unit, Papageorgiou General Hospital, 56403 Thessaloniki, Greece; arvanitik@hotmail.com; 3 Department of Radiology, Papageorgiou General Hospital, 56403 Thessaloniki, Greece; christospapastergiou65@gmail.com

**Keywords:** fever of unknown origin, FUO, PET/CT, ^18^F-FDG-PET/CT

## Abstract

Conventional diagnostic imaging is often ineffective in revealing the underlying cause in a considerable proportion of patients with fever of unknown origin (FUO). The aim of this study was to assess the diagnostic value of fluorine-18 fluorodeoxyglucose positron emission tomography/computed tomography (^18^F-FDG-PET/CT) in patients with FUO. We retrospectively reviewed ^18^F-FDG-PET/CT scans performed on 50 consecutive adult patients referred to our department for further investigation of classic FUO. Final diagnosis was based on histopathological and microbiological findings, clinical criteria, or clinical follow-up. Final diagnosis was established in 39/50 (78%) of the patients. The cause of FUO was infection in 20/50 (40%), noninfectious inflammatory diseases in 11/50 (22%), and malignancy in 8/50 (16%) patients. Fever remained unexplained in 11/50 (22%) patients. ^18^F-FDG-PET/CT scan substantially contributed to the diagnosis in 70% of the patients, either by identifying the underlying cause of FUO or by directing to the most appropriate site for biopsy. Sensitivity, specificity, accuracy, positive predictive value (PPV) and negative predictive value (NPV) of ^18^F-FDG-PET/CT for active disease detection in patients with FUO were 94.7%, 50.0%, 84.0%, 85.7%, and 75.0%, respectively. In conclusion, whole-body ^18^F-FDG-PET/CT is a highly sensitive method for detection of the underlining cause of FUO or for correctly targeting suspicious lesions for further evaluation.

## 1. Introduction

Despite the immense progress of laboratory and imaging modalities, fever of unknown origin (FUO) remains a diagnostic challenge. FUO was originally defined by Petersdorf and Beeson in 1961 as body temperature higher than 38.3 °C, on at least three occasions over a period of at least three weeks, with no diagnosis made despite one week of inpatient investigation [1]. The initial definition of FUO was subsequently modified by Durack and Street in 1991 by removing the requirement of inpatient investigation and also by excluding immunocompromised patients as they may require an entirely different diagnostic approach [2]. Later, the quantitative criterion of uncertain diagnosis after a period of time was proposed to be replaced by a qualitative criterion of a number of obligatory investigations that should be performed to qualify the condition as FUO [3,4,5].

The differential diagnosis of FUO includes a wide spectrum of highly heterogeneous diseases, which is traditionally subdivided into four categories: infections, malignancies, non-infectious inflammatory diseases (NIID), and miscellaneous causes, with their incidence strongly affected by the local epidemiology [6,7]. Expectedly, the proportion of undiagnosed cases of FUO ranged from 7% to 53% in various studies, thus indicating that the diagnostic investigation of FUO still remains a challenge [7,8].

Structural cross-sectional imaging modalities such as computed tomography (CT) and magnetic resonance imaging (MRI) can be used to detect focal pathologies, but they may be less accurate in the early stages of infectious and inflammatory diseases. Furthermore, distinction of active inflammation from healing or treated infection or postoperative changes and maturing scar tissue is often hardly achievable by radiological modalities [9].

Conversely, nuclear medicine modalities are capable of early detection of disease activity at a cellular or even molecular level, preceding morphological alterations, and also to distinguish between active and inactive disease and between infection and aseptic inflammation or malignancy [10]. In the few past decades, a variety of specific and non-specific radiopharmaceuticals have been proposed for imaging infection and inflammation [11]. ^67^Ga (gallium) citrate scintigraphy has been widely used in the past to investigate FUO [12,13,14] due to its accumulation in both infections and acute or chronic inflammations and neoplasms. However, its low specificity and suboptimal imaging characteristics, along with the introduction of newer radiopharmaceuticals for imaging of infection and inflammation like labeled leucocytes and ^18^F-fluorodeoxyglucose (^18^F-FDG) have dramatically reduced its use in most clinical indications, including FUO.

Labeled leucocyte scintigraphy is a highly specific method for imaging infection because labeled leucocytes migrate actively into infectious foci [15]. However, it is not a very helpful modality in patients with FUO, because infections account for only a portion of FUO cases, ranging from 11% to 57% in various studies [6,7,8].

^18^F-FDG, the most commonly used radiotracer for positron emission tomography/computed tomography (PET/CT), accumulates avidly in most viable neoplasms and has been extensively studied in patients with malignancies for diagnosis, staging, and treatment response assessment [16]. Since Tahara et al. first showed high ^18^F-FDG uptake in abdominal abscesses in 1989 [17], evidence is growing on the usefulness of ^18^F-FDG-PET/CT in the diagnosis and management of several inflammatory and infectious diseases [18] based on the high glucose uptake by activated inflammatory cells, related to their increased glycolytic activity and overexpression of glucose transporters (GLUT), especially GLUT 1 and GLUT 3. Shorter procedure duration and higher resolution and sensitivity are the comparative advantages of ^18^F-FDG-PET over conventional scintigraphy, making ^18^F-FDG-PET an appealing modality for imaging infection and inflammation especially since the emergence of PET/CT [19].

Several studies indicate the potential contributory role of both ^18^F-FDG-PET alone [12,13,20,21,22,23] and ^18^F-FDG-PET/CT [24,25,26,27,28] in the management of patients of FUO. However, the representativeness of the populations studied may be questionable as in the majority of the Northwestern European studies NIID is the leading causes of FUO, whereas in those coming from Asia, infections are more common, with tuberculosis predominating. As far as we know, only limited data are available regarding Southern Europe and Mediterranean countries. For example, to the best of our knowledge, there are only two previous studies on FUO in Greece [29,30], none of them dealing with the role of ^18^F-FDG-PET/CT imaging in the diagnosis of FUO.

We performed a retrospective study at a tertiary academic general hospital in Northern Greece in order to assess the diagnostic value of ^18^F-FDG-PET/CT in patients presenting with FUO.

## 2. Experimental Section

### 2.1. Patient Population

Fifty consecutive immunocompetent adult patients of Caucasian origin were studied retrospectively. Patients were admitted to the PET/CT department of Papageorgiou General Hospital in Northern Greece, between November 2016 and July 2019, for further classic FUO investigation.

All patients enrolled in the study fulfilled the revised Petersdorf’s criteria of FUO [3]. Patients with nosocomial infections or known immunodeficiency (e.g., neutropenia, HIV-associated infection, hypogammaglobulinemia or on systemic corticosteroids) were excluded from the study.

The initial diagnostic work-up of all patients included a comprehensive medical history, physical examination, routine hematological, biochemical, and serological tests, blood and urine cultures and plain chest radiographies. Concerning the inflammatory blood markers, values higher than 20 mm/h, 0.8 mg/dL, and 0.5 ng/mL were considered as indicative of abnormally elevated erythrocyte sedimentation rate (ESR), C-reactive protein (CRP) level, and procalcitonin (PCT), respectively. Computed tomography (CT), MRI and echocardiography had been performed in the vast majority of the patients prior to ^18^F-FDG-PET/CT imaging, while invasive investigations such as endoscopies and parenchymal organs, bone marrow, or temporal artery biopsies had been occasionally conducted.

Underlying pathologies that could be related to the cause of the fever or might affect the interpretation of the ^18^F-FDG-PET/CT scan were recorded for all patients. The presence of diabetes mellitus along with relevant blood glucose lowering drugs was recorded. Antibiotic or corticosteroid treatment prior to ^18^F-FDG-PET/CT imaging or chemotherapy in the last 6 months was also recorded.

### 2.2. ^18^F-FDG-PET/CT Imaging

All ^18^F-FDG-PET/CT scans were performed using a 16-slice integrated PET/CT scanner (Discovery 710; GE Healthcare).

Patients fasted for at least 12 h before intravenous injection of ^18^F-FDG at a dose of 4 MBq/kg of body weight. In patients carrying cardiovascular implantable electronic devices (CIED) or prosthetic cardiac valves, suspected of cardiac infection as the cause of FUO, a preparation protocol for suppression of myocardial glucose metabolism was applied, consisting of a high fat–low carbohydrate diet started three days before imaging followed by a prolonged (≈18 h) fasting. The target serum glucose levels at the time of ^18^F-FDG administration was less than 150 mg/dL.

Skull base to mid-thigh PET/CT imaging started 60 min after intravenous injection of ^18^F-FDG, at a 3 min per bed position rate in a three-dimensional mode. On clinical suspicion of the involvement of lower extremities, a whole-body scan including the legs was performed. Delayed regional images were additionally obtained in cases of ambiguous findings. Low-dose helical CT without contrast enhancement (30–300 mA automatically adjusted to tissue depth, 120 kV, slice thickness of 3.75 mm) was performed for attenuation correction of PET emission data and anatomic mapping.

PET sections were obtained by an iterative reconstruction algorithm (ordered subset expectation maximization (OSEM)) and corrected for attenuation by the corresponding CT attenuation maps. Maximum intensity projection (MIP) images and reconstructed sections (low-dose CT, attenuation corrected PET, and fused PET/CT) were then displayed for analysis in the standard axial, coronal, and sagittal planes.

### 2.3. Image Analysis

All ^18^F-FDG-PET/CT images were reviewed by two experienced nuclear medicine physicians and a radiologist aware of the clinical data. Disagreement between the readers were resolved by consensus. Image interpretation was based on visual inspection of the body for areas of abnormally high ^18^F-FDG uptake. In addition, in cases of hypermetabolic PET foci adjacent to hyperdense CT findings (e.g., prosthetic cardiac valves or heavy coronary vessel calcification), the non-attenuation corrected (NAC) sections were thoroughly inspected, to exclude PET false positivity resulting from attenuation overcorrection.

Studies were considered as positive for active disease if increased ^18^F-FDG uptake, focal or diffuse, other than normal or otherwise explainable was observed. The pattern (focal, linear, or diffuse) and the intensity of ^18^F-FDG uptake were visually assessed. Conversely, studies with a normal or otherwise explainable ^18^F-FDG pattern of distribution throughout the body were classified as negative for active disease processes.

A positive study was classified as “true positive” (TP) when abnormal ^18^F-FDG uptake in an organ or tissue corresponded to the cause of fever, as confirmed by additional investigations, and as “false positive” (FP) when it was proven unrelated to the cause of fever or when the fever remained undiagnosed during the follow-up period.

A negative study was classified as “true negative” (TN) when no cause of fever was identified during the clinical follow-up for at least 6 months or the fever resolved spontaneously without specific treatment, and as “false negative” (FN) when a focal infection, inflammation, or malignancy was eventually identified as the cause of fever within 6 months or fever persisted throughout the follow-up period or the patient died febrile without a definite diagnosis.

Follow-up was accomplished by reviewing the patients’ medical records or by contacting the referring physician or the patients themselves.

Final diagnosis was based on histopathological and microbiological findings, on fulfillment of widely acceptable diagnostic criteria or clinical follow-up. The duration of follow-up exceeded 6 months in all patients without a definite diagnosis.

An ^18^F-FDG-PET/CT scan was considered as contributory to the diagnosis if it directly identified the underlying cause of FUO or correctly suggested the site for a diagnostic biopsy. In all other situations, it was considered as non-contributory to the diagnosis.

^18^F-FDG uptake by suspect lesions was also semi-quantitatively evaluated by means of the maximum standardized uptake value (SUVmax). SUVmax was derived using properly sized spherical volumes of interest (VOIs) according to current EANM (European Association of Nuclear Medicine) guidelines [31]. In case of multiple hypermetabolic foci, the highest relevant SUVmax value was recorded.

### 2.4. Statistical Analysis

Sensitivity, specificity, accuracy, positive predictive value (PPV), and negative predictive value (NPV) of ^18^F-FDG-PET/CT scan for the detection of active disease were calculated as per standard definitions. Continuous variables were expressed either as means ± standard deviation (SD), or as medians and interquartile range, as appropriate. Categorical variables were expressed as number and proportions and the between-groups differences were tested by means of Pearson’s X^2^ test (or Fisher’s exact test where applicable). Differences of the continuous variables between patient groups were tested for significance using either *t*-test for normally distributed variables or the nonparametric Mann–Whitney U tests as appropriate. Statistical significance was accepted for *p* < 0.05. Statistical analysis was accomplished using the IBM SPSS 23.0 statistic software package (IBM Corp., Armonk, NY, USA).

## 3. Results

From November 2016 to July 2019, fifty-four patients were referred to our PET/CT facility installed in a 700-bed academic general hospital, for classic FUO investigation. The majority of the patients were mainly coming from the Internal Medicine or Infectious Diseases departments of other hospitals in the area. Four patients were excluded from the study; one was 16 years old, two were lost to follow-up, and one with an ^18^F-FDG-PET/CT scan highly suspicious for lymphoma who died shortly after without a definite diagnosis. Thus, 50 adult patients all having ^18^F-FDG-PET/CT scan for classic FUO investigation were eventually included in the study.

### 3.1. Patients Characteristics and Final Diagnoses

The main demographic and clinical characteristics of the patients enrolled in the study are summarized in Table 1.

Because of the varying origin of the patients and the retrospective nature of the study, a uniform diagnostic work-up before ^18^F-FDG-PET/CT imaging was missing. However, after the initial diagnostic work-up and before ^18^F-FDG-PET/CT scan, almost all of them (49/50) had been submitted to several advanced investigations (median 3, min–max 0–8). The advanced investigations performed prior to the ^18^F-FDG-PET/CT scan on our patients are listed in Table 2.

A final diagnosis was established in 39/50 (78%) patients and was classified into 4 categories: infection, malignancy, non-infectious inflammatory diseases (NIID), and undiagnosed fever. The cause of FUO was infection in 20 patients (40%), malignancy in 8 patients (16%), NIID in 11 patients (22%), while the fever remained unexplained in 11 patients (22%). The final diagnoses for the 50 patients studied are listed in Table 3.

### 3.2. ^18^F-FDG-PET/CT Results

The standard preparation protocol for ^18^F-FDG-PET/CT imaging was applied to 45 of the 50 patients studied, whereas five patients successfully followed the preparation protocol for cardiac imaging. Among them, in 4 patients, the fever was found unrelated to cardiac infection, while in one patient the cause of fever was CIED associated infection; however, in this patient, the ^18^F-FDG-PET/CT scan was false negative.

Mean serum glucose levels of the patients at the time of ^18^F-FDG administration was 96.7 ± 20.2 mg/dL (min–max 63–155 mg/dL) and did not differ between patients with contributory and non-contributory scans (94.8 ± 17.8 mg/dL vs. 101.3 ± 25.1 mg/dL), respectively (*p* = 0.077).

^18^F-FDG-PET/CT scan was abnormal in 42/50 (84%) patients studied, showing single or multiple hypermetabolic foci compatible with active disease, while the scan was negative for active disease in 8 patients (16%).

Of the 42 positive ^18^F-FDG-PET/CT scans, 36 were considered as true positive (TP) scans and 6 as false positive (FP) scans. Thus, a definite diagnosis was established in 85.7% of patients with positive scans. The TP scans included 19 cases of infections, 8 cases of malignancy, and 9 cases of non-infectious inflammatory diseases. The TP scans in the group of infections included all the cases of infectious diseases listed in Table 3, except of one case of CIED-associated infection, in which the ^18^F-FDG-PET/CT scan was false negative.

All the 8 patients with a final diagnosis of malignancy (5 newly diagnosed non-Hodgkin’s lymphomas, 1 Hodgkin’s disease, 1 lung cancer, and 1 urinary tract carcinoma relapse) had a true positive ^18^F-FDG-PET/CT scan. Among them, there was only one with recurrence of a previous malignancy (recurrence of urinary tract carcinoma initially diagnosed 4 years ago) and another with aggressive transformation of a previous hematological malignancy (Waldenstrom macroglobulinemia diagnosed 5 years ago, now diagnosed with non-Hodgkin’s lymphomas). Of the 6 other patients, 5 had no history of malignancy, and 1 had a history of a different malignant disease (breast cancer diagnosed 6 years ago).

The 9 TP scans in the group with NIID included three patients with large vessel vasculitis and one of each of the following: sarcoidosis, polymyalgia rheumatica, familial Mediterranean fever, adult-onset Still’s disease, subacute thyroiditis, and exacerbation of inflammatory bowel disease.

There were 6 FP scans; they included 4 cases of undiagnosed fever with spontaneous resolution during the follow-up period, one case of adult-onset Still’s disease, and a case of neo-esophagus inflammation from gastroesophageal reflux.

Eight out of fifty patients studied had a negative ^18^F-FDG-PET/CT scan. Six of them were considered true negative (TN); in five of these cases the fever resolved spontaneously with no evidence of disease during the at least 6-month follow-up period, while in one case the fever resolved after corticosteroid administration. Finally, there were two false negative (FN) scans; the first case was an elderly patient with recurrent febrile episodes until death a year later with a possible diagnosis of viral encephalitis and the second one was a febrile patient who was eventually diagnosed, according to clinical criteria and echocardiography, with CIED-associated infection, whose fever resolved after the CIED removal. The last patient was on antibiotic treatment for prostatitis for two weeks before the ^18^F-FDG-PET/CT scan without remission of the fever. Thus, among the patients with negative scans, a definite diagnosis was established in only one (12%).

^18^F-FDG-PET/CT results according to the category of final diagnosis are depicted in Table 4. Nineteen of twenty patients in the group of infections had a true positive scan. The final diagnoses in patients with false (positive or negative) ^18^F-FDG-PET/CT scans are presented in Table 5.

**Figure 1:**
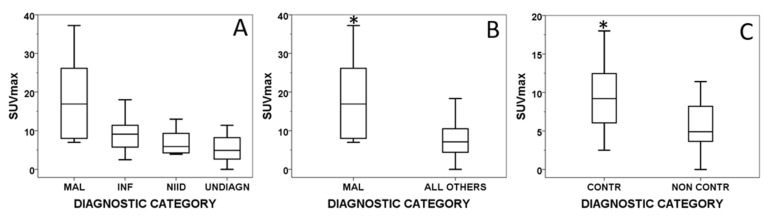
Box plot graphs showing SUVmax distribution in various diagnostic categories. Across the four groups of final diagnosis (**A**); between malignancy and non-malignancy (**B**) and between contributory and non-contributory scans (**C**). SUVmax, maximum-standardized uptake value; NIID, non-infectious inflammatory disease; MAL, malignant; INF, infectious; NIID, non-infectious inflammatory disease; UNDIAGN, undiagnosed; CONTR, contributory; NONCONTR, non-contributory; * *p* < 0.05.

The median SUVmax [IQR] was higher in malignant diseases (16.9 [18.7]) followed by that in infections (9.1 [6.1]), NIID (6.2 [6.6]) and in undiagnosed fever (5.9 [6.3]). The median SUVmax [IQR] was significantly higher in malignant diseases than in all the other diagnoses together (16.9 [18.7] vs. 7.1 [6.2], *p* = 0.01). Similarly, the median SUVmax [IQR] was significantly higher in contributory than in non-contributory ^18^F-FDG-PET/CT scans (9.2 [7.1] vs. 4.9 [5.7], *p* = 0.01). ^18^F-FDG uptake quantified by SUVmax in different groups of final diagnosis is graphically presented in Figure 1.

The overall sensitivity, specificity, accuracy, positive predictive value (PPV), and negative predictive value (NPV) of ^18^F-FDG-PET/CT for active disease detection in our patients were 94.7%, 50.0%, 84.0%, 85.7%, and 75.0%, respectively. The sensitivity of ^18^F-FDG-PET/CT for diagnosing active disease processes was higher in the group of malignancies where all of the scans were true positive (sensitivity of 100%) followed by the group of infections where the ^18^F-FDG-PET/CT scans were true positive in 19/20 patients and false negative in 1/20, giving a sensitivity of 95%. However, due to the relatively small number of patients in the different groups of diagnoses, no further analysis of the diagnostic performance of ^18^F-FDG-PET/CT in each group of patients was undertaken.

The ^18^F-FDG-PET/CT scan was considered contributory to the diagnosis in 35/50 (70%) of the patients, either by identifying the underlying cause of FUO (causal diagnosis in 25 patients) or by correctly directing to the most appropriate site for successful biopsy leading to an accurate diagnosis (biopsy site selection in 10 patients). All the TP ^18^F-FDG-PET/CT scans, but one, were considered as contributory to the diagnosis, and this was the case of a patient with a true positive scan but diagnosis of adult-onset Still’s disease based on exclusion criteria of other diseases, thus allocating the PET/CT scan to the not contributory to the diagnosis category. The true negative ^18^F-FDG-PET/CT scans as well as the false positive and false negative scans were considered as non-contributory to the diagnosis.

**Figure 2:**
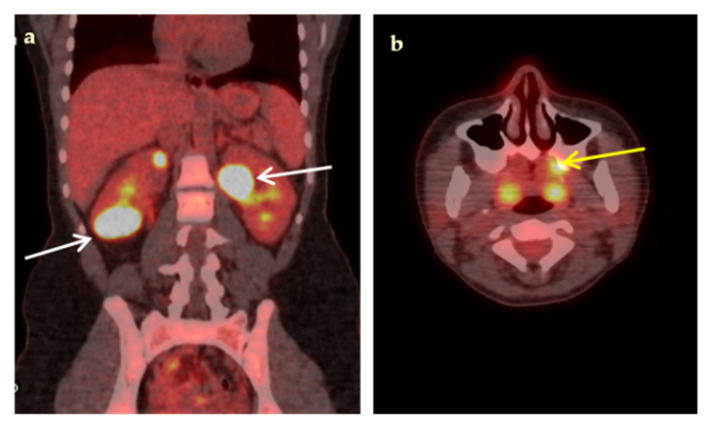
An 18-year-old woman presented with a 3-week fever, elevated inflammatory blood markers (ESR 77 mm/h, CRP 9.3 mg/dL) and an abdominal CT scan suggestive of possible renal abscesses. Coronal fused ^18^F-FDG-PET/CT image (**a**), demonstrated multiple hypodense, highly hypermetabolic lesions in both kidneys, the largest (white arrows) in the lower pole of the right kidney (SUVmax 35.3) and in the upper pole of the left kidney (SUVmax 34.4), compatible with renal abscesses, confirmed by biopsy. Transaxial fused ^18^F-FDG-PET/CT image (**b**) demonstrated high ^18^F-FDG paradental uptake in the left maxilla (yellow arrow) raising concern for hematogenous spread of dental infection. Complementary focused interrogation revealed a history of a painful, undertreated dental condition of the left maxilla preceding fever.

**Figure 3:**
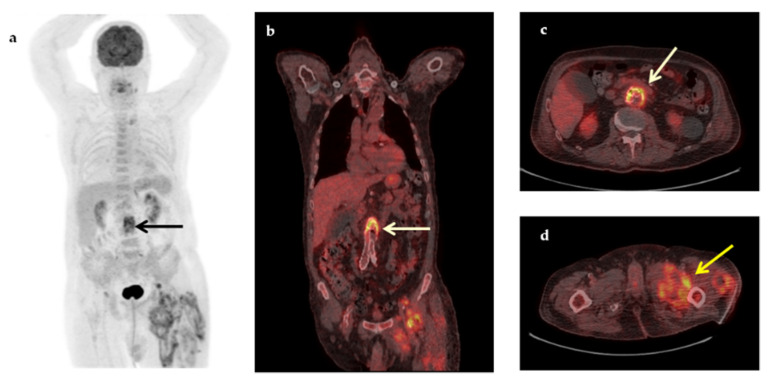
A 74-year-old man with a medical history of aortobiiliac vascular prosthesis because of an asymptomatic aneurysm 5 months ago, presented with a 2-month fever, increased inflammatory blood markers (ESR 83 mm/h, CRP 19.2 mg/dL) and intramuscular fluid collections in the left thigh, (revealed in a CT for localized pain). Maximum intensity projection ^18^F-FDG-PET (**a**), coronal fused (**b**) and transaxial fused ^18^F-FDG-PET/CT images at the level of the L3 vertebra (**c**), demonstrated increased metabolic activity in the wall of the abdominal aneurysm (arrows) at that level (SUVmax 7.0), suspicious of infection. In addition, transaxial fused FDG-PET/CT image at the level of thighs (**d**) revealed intramuscular hypermetabolic collections with air bubbles in the left thigh, suspicious of abscesses (yellow arrow). Vascular graft infection was confirmed by histopathology after removal of the aortic graft (*Klebsiella pneumoniae*, *Pseudomonas aeruginosa*).

**Figure 4:**
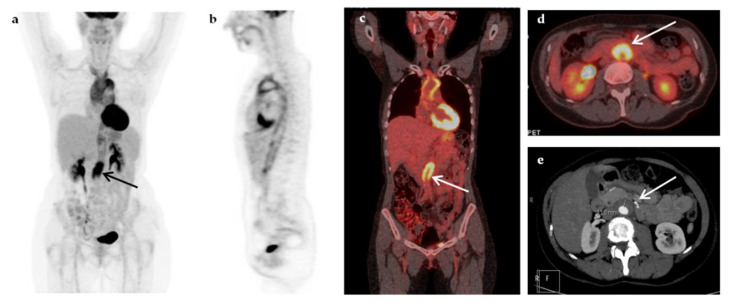
A 52-year-old woman presented with prolonged fever (over 6 months) and increased inflammatory blood markers (ESR 76 mm/h, CRP 9.3 mg/dL). The patient had a medical history of breast cancer seven years ago. Maximum-intensity projection ^18^F-FDG-PET (**a**), sagittal ^18^F-FDG-PET (**b**), coronal fused ^18^F-FDG-PET/CT (**c**), and transaxial fused FDG-PET/CT images at the level of the renal vessels (**d**) demonstrated increased metabolic activity within the wall of the thoracic and abdominal aorta, extending to the subclavian arteries, more intense at the root of the aorta and above the aortic bifurcation (arrows). The findings were compatible with vasculitis of large- and medium-sized arteries, mainly affecting the aorta (panaortitis). Transverse section of the CT angiography at the level of renal vessels (**e**) showed thickening of the aorta wall, more pronounced at the level below the renal vessels (arrow), but with no evidence of narrowing of the aortic lumen. A diagnosis of large vessel vasculitis/Takayasu’s arteritis was made, and fever resolved after treatment with corticosteroids.

**Figure 5:**
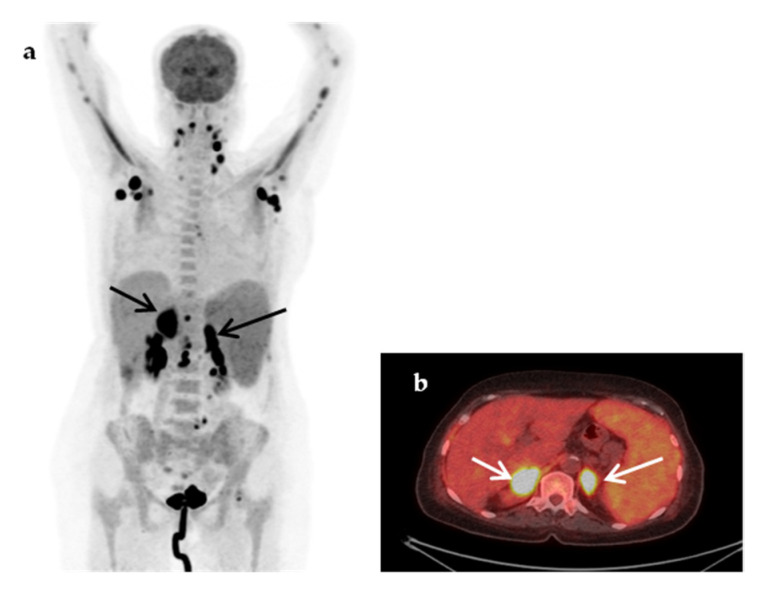
A 75-year-old woman presented with a 4-week fever associated with malaise and weight loss. The abdominal CT revealed splenomegaly and adrenal masses. Maximum-intensity projection (MIP) ^18^F-FDG-PET (**a**) showed extensive highly hypermetabolic lymphadenopathy, cervical, axillary (SUVmax 22.7) and abdominal and hypermetabolic (SUVmax 16.8) adrenal masses (fused transaxial ^18^F-FDG-PET/CT image (**b**), arrows), hypermetabolic hepatic lesions (at least two) and multiple hypermetabolic bone metastases and splenomegaly with diffuse homogeneously increased metabolic activity. The findings were suspicious of lymphoma. A diagnosis of non-Hodgkin’s lymphoma was confirmed by histopathology after biopsy of an axillary lymph node.

**Figure 6:**
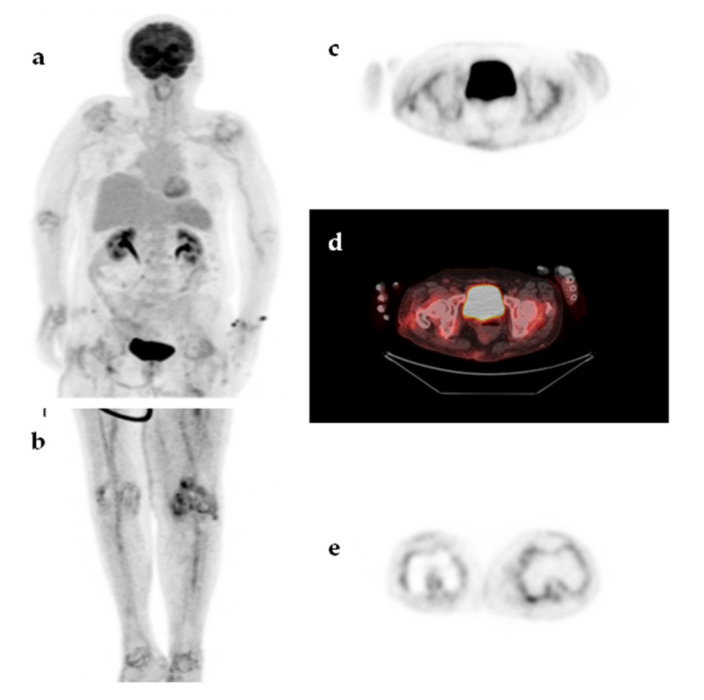
An 85-year-old woman presented with a 2-month fever. The patient had a history of total left knee arthroplasty with no signs of loosening or infection. MIP ^18^F-FDG-PET image (**a**,**b**), transaxial ^18^F-FDG-PET and ^18^F-FDG-PET/CT images at the level of hips (**c**,**d**) and transaxial ^18^F-FDG-PET image at the level of knees (**e**) demonstrated diffuse, symmetric, moderately increased ^18^F-FDG uptake, in the large peripheral joints (shoulders, hips, knees) accompanied by increased ^18^F-FDG uptake along the medium-sized arteries (axillary, humeral, femoral, and tibial arteries). The findings were compatible with polymyalgia rheumatica and the fever resolved upon treatment with corticosteroids.

Some representative cases of the diagnostic contribution of ^18^F-FDG-PET/CT scan in patients with FUO are shown in Figure 2, Figure 3, Figure 4, Figure 5 and Figure 6.

### 3.3. Baseline Patient Characteristics in Contributory and Non-Contributory Scans

A detailed comparison of many clinical (age, gender, fever duration, prior antibiotic administration, lymphadenopathy, splenomegaly, presence of diabetes mellitus) and laboratory (number of prior advanced investigations and levels of elevated inflammatory blood markers) characteristics showed no significant differences between the patients with contributory and non-contributory ^18^F-FDG-PET/CT scans (Table 6).

In particular, we did not find any significant difference of the duration of the fever before the ^18^F-FDG-PET/CT scan between patients with a contributory and those with a non-contributory ^18^F-FDG-PET/CT scan (median fever duration 30 (21–365) days vs. (30–330) days respectively (*p* = 0.08).

Increased inflammatory blood markers including erythrocyte sedimentation rate (ESR), C-reactive protein (CRP), or procalcitonin (PCT)) were recorded in 31 patients. An increase of CRP level, in particular, was recorded in 22 of the patients studied, with a mean value of 10.9 ± 9.8 mg/dL. All patients had a positive ^18^F-FDG-PET/CT scan (20 true positive and 2 false positive). Among the 20 patients with increased CRP levels and true positive ^18^F-FDG-PET/CT scans the final diagnosis was infection in 13 (65%), NIID in 4 (20%), and malignancy in 3 (15%). In the two patients with increased CRP level and a false positive ^18^F-FDG-PET/CT scan, no diagnosis was established, and the fever resolved spontaneously in one patient and after steroid administration in the other.

## 4. Discussion

Establishing a diagnosis for FUO remains challenging. ^18^F-FDG, as a non-specific indicator of increased glycolytic metabolism, is concentrated not only in infectious sites but also in NIID and in neoplasms, all being possible causes of FUO. Several studies support the use of ^18^F-FDG-PET in the assessment of FUO [12,13,20,21,22,23,24,25,26,27,28,32,33,34,35,36,37]. Moreover, an abnormal ^18^F-FDG-PET/CT scan, as part of a structured diagnostic protocol for FUO, has been shown to be among the significant predictors for reaching a diagnosis [32].

The present study assessed the diagnostic value of ^18^F-FDG-PET/CT in 50 consecutive, non-immunocompromised, adult patients with FUO referred in a tertiary academic general hospital in Northern Greece. A definite diagnosis was established in 78% of our patients with infections being identified as the leading cause of FUO (40%). The percentage of patients diagnosed with infections in the present study was higher compared to those in studies coming from Northwestern Europe where NIID accounted for the most cases of FUO [4,5,20,21,32], but similar to the results of an older Central European study [12] and two recent Asian studies [27,36] where infections accounted for the most cases of FUO. However, in contrast to Asian studies, where tuberculosis was the most common infectious cause of FUO, only 2 of 20 cases of infection were due to tuberculosis in our study, probably reflecting differences in the degree of disease control among countries worldwide.

Non-infectious inflammatory diseases commonly constitute a major FUO contributor in developed countries. In the present study, NIID was the second leading cause of FUO (22%), with large-vessel vasculitis being the most common cause in this group of patients. The high diagnostic yield of ^18^F-FDG-PET/CT in detecting active large-vessel vasculitis (LVV) has been convincingly shown [20,28,33,35], and the investigation of patients suspected for LVV is currently among the major non-oncological indications of ^18^F-FDG-PET/CT [38].

The percentage of patients diagnosed with malignancies in our study was quite low (16%), similar to that observed in many previous studies [21,27,28,32], which could be explained by the widespread early use of cross-sectional imaging (ultrasound CT, MRI) resulting in a reduction of cases of malignancies presented as FUO. Non-Hodgkin’s lymphoma was the most common malignant cause of FUO in our study, as in previous studies [27,35,36]. Notably, the ^18^F-FDG-PET/CT scan was true positive in all patients with proven malignancy, thus, contributing to the diagnosis by directing toward a confirmatory biopsy.

In the present study, fever remained undiagnosed in 11 (22%) of patients. The proportion of patients with undiagnosed fever varies widely in the literature, ranging from 7% to 53% [4,8,20,24,25,26,27,28,32,33]. This variation may be due to differences in local public health status, availability of advanced imaging techniques and timing of ^18^F-FDG-PET/CT examination. In our study, the percentage of undiagnosed cases was on average comparable to or even lower than that of previous published studies, suggesting a rather early use of ^18^F-FDG-PET/CT in our patients. Earlier application of ^18^F-FDG-PET/CT in the diagnostic algorithm could facilitate the early diagnosis, reducing the number of unnecessary tests and the duration of hospitalization and could be cost-effective [39,40,41]. In 7 undiagnosed patients (64%) of our study, the fever resolved spontaneously during the follow-up period. Spontaneous remission of the fever is common in patients with longstanding undiagnosed classic FUO [4,7,42]. In a recent meta-analysis of 13 studies including approximately 550 patients with classic FUO a negative ^18^F-FDG-PET/CT scan after a series of unsuccessful investigations for fever workup, was associated with high likelihood of spontaneous remission [43].

Comparing our results with the two previous studies in the Greek population, some interesting points emerged. In the first Greek study published in 2010, including 112 patients, the leading causes of FUO were NIID followed by infections and malignancies (33%, 30.4%, and 10.7%, respectively) whereas the undiagnosed cases were 20.5% [29]. In our study, the proportion of NIID was lower (22%), with the leading causes of FUO being the infectious diseases, mainly abdominal infections. Coming to the present, our findings are in accordance with that of a recent (2019) Greek study including 48 patients, showing a distribution similar to ours of the causes of FUO, with infections being the most common causes (29.2%), followed by NIID (25%) and malignancies (16.6%) [30]. This apparent shift of FUO causation in Greece over time toward the infectious part of the list, may be multifactorial. Increasing frequency of aggressive interventions (vascular or gastroenterological stenting, implantable devices, etc.) in combination with the epidemic of microbial resistance to antibiotics and the impact of the recent economic crisis on infectious disease transmission and control could be some reasonably explanatory candidates [44]. Finally, the percentage of undiagnosed cases in this geographic area did not significantly change over the last ten years (ranging from 20.5% to 25% in the 3 studies) irrespective of the addition of ^18^F-FDG-PET/CT in the diagnostic sequence, an observation potentially suggestive of the existence of a non-imageable subset of conditions among the causes underlying FUO.

The overall sensitivity and specificity of ^18^F-FDG-PET/CT for active disease detection calculated in our study were 94.7% and 50.0%, respectively, in accordance with two recent meta-analyses supporting the diagnostic role of ^18^F-FDG-PET/CT in patients with FUO. The first of them published in 2016, including 42 studies with 2058 patients with FUO reported a pooled sensitivity and specificity of ^18^F-FDG-PET/CT of 86% and 52%, respectively [45]. The second one, published in 2018, including 23 studies with 1927 patients concluded that ^18^F-FDG-PET/CT was very helpful for recognizing and for excluding, as well, diseases as causes of FUO with a pooled sensitivity and specificity of 84% and 63%, respectively [39]. The sensitivity of ^18^F-FDG-PET/CT in our study was higher in the group of malignancies where all of the scans were true positive reaching a sensitivity of 100%, followed by the group of infections with true positive scans in 95% of patients, missing only one case of infectious disease. This was in agreement with previous studies highlighting the superior clinical efficacy of ^18^F-FDG-PET/CT in populations with higher proportions of patients with infections and malignancies [27,28,37,45].

However, in the setting of FUO, comparison of different studies in terms of sensitivity and specificity may be misleading for a number of reasons, including variation in FUO definition, patient characteristics, diagnostic work-up sequence, and diagnostic gold standard multiplicity. In an attempt to overcome these problems, the estimation of the clinical helpfulness of PET scan in the diagnosis of FUO has been suggested instead of the formal sensitivity and specificity [10]. During the last two decades, several studies have explored the diagnostic contribution of stand-alone ^18^F-FDG-PET [12,13,20,21,22,23] and more recently of the ^18^F-FDG-PET/CT scans [24,25,26,27,28,33,34,35,36] in patients of FUO, concluding clinical helpfulness varying widely between 16% and 69% for the stand-alone ^18^F-FDG-PET studies and between 38% and 75% for the ^18^F-FDG-PET/CT studies.

In the present study, ^18^F-FDG-PET/CT was helpful and substantially contributed to the diagnosis in 70% of patients, either by identifying the underlying cause of FUO or by correctly targeting suspicious lesions for diagnostic biopsy. Only the true positive scans were considered as contributory to the diagnosis in our study. All the other scans, including the true negative ones were considered as non-contributory to the diagnosis. Similarly, the majority (12/14) of studies included in a recent meta-analysis [40] considered only the positive ^18^F-FDG-PET and ^18^F-FDG-PET/CT scans as helpful to the diagnosis. However, this approach has been questioned, as in a recent meta-analysis of 13 studies [43] it was concluded that the diagnostic yield of ^18^F-FDG-PET/CT in patients with FUO should take into account not only the positive cases but also the true negative ones claiming that patients with a negative ^18^F-FDG-PET/CT scans are more likely to have a favorable course. Although it may be true for a considerable fraction of patients with undiagnosed fever, we have not allocated the true negative scans to the contributory to the diagnosis ones in our study, because a negative ^18^F-FDG-PET/CT scan did not actually explain the cause of the fever, which may remain virtually undiagnosed until its, often spontaneous, remission.

In our study, a definite diagnosis was established in a high percentage of patients with positive scans (PPV of 85.7%), in accordance with previous studies [12,20,23,24,35]. A meta-analysis of 14 studies showed that an abnormal ^18^F-FDG-PET scan is associated with increased likelihood of definite diagnosis, thus, favoring the adoption of ^18^F-FDG-PET as a first-line investigation in FUO [40]. On the other hand, in our study, a definite diagnosis was established in a very low percentage of patients with negative scans, in particular in only one out of the 8 patients with negative scans (12%). A presumptive diagnosis could explain the fever in another case while the remaining 6 cases with negative scans were considered as true negatives given a high enough NPV of 75%. High NPV of ^18^F-FDG-PET or ^18^F-FDG-PET/CT in the context of FUO had also been steadily reported in previous studies [12,20,23,24].

Regarding patient characteristics tested for a possible correlation with contributory PET/CT scans, in contrast to previous studies [4,27,32,33,36], we did not find any significant differences in any of the variables tested (age, gender, prior antibiotic administration, presence of lymphadenopathy or splenomegaly, presence of diabetes mellitus, number of advanced diagnostic tests performed before PET/CT scan, serum glucose level at the time of ^18^F-FDG administration, or inflammatory blood markers) between patients with contributory and not contributory scans.

In particular, the duration of the fever before the ^18^F-FDG-PET/CT scan, did not differ significantly in our study between patients with contributory and non-contributory ^18^F-FDG-PET/CT CT scans, although the duration of the fever was shorter in patients with contributory scans. This finding might be in contrast with those of previous studies [4,27,32,36] reporting a positive correlation between short fever duration and a positive scan.

Many studies investigating the clinical value of ^18^F-FDG-PET/CT scan in patients with FUO have already reported an elevated CRP as a significant predictor for a positive scan [21,25,32,33,36,46]. In accordance with these studies, an increased CRP level was observed in 22 patients; all of them had a positive ^18^F-FDG-PET/CT scan, which was true positive in 90.9% of them. In fact, most of these patients (65%) were eventually diagnosed with infection.

In contrast to its established use in oncology, the standardized uptake value (SUV) has not been adequately assessed as a semi-quantitative measure of the severity of inflammation and infection and there is no cutoff value suggested to avoid false positive results [16]. Therefore, its calculation in infection and inflammation is not a standard practice, although it may be helpful in repeated studies for response to treatment. In our study, the SUVmax value was significantly higher in malignancies than in all the other diagnoses. This was not a surprise as SUV values are generally higher in malignant lesions compared to benign lesions. In a large Chinese multi-center retrospective study including 376 patients with FUO and inflammation of unknown origin (IUO) [47], ^18^F-FDG uptake, estimated by either SUVmax or by visual inspection scoring was significantly higher in malignant compared to non-malignant diseases. Moreover, a significantly higher SUVmax in the contributory scans compared to the non-contributory ones was observed in our study. Our findings on SUVmax, taken together, suggest that a high SUVmax found in the context of FUO investigation may be indicative of underlying malignancy as a cause of FUO and may be also associated with a better diagnostic performance of the ^18^F-FDG-PET/CT scan.

The main limitation of the present study is its retrospective nature, closely associated with the lack of a uniform diagnostic work-up before ^18^F-FDG-PET/CT imaging. Both the diagnostic tests performed prior to ^18^F-FDG-PET/CT scan, and the timing of the scan itself, were conducted at the discretion of the referring physicians, widely varying among the patients. Nevertheless, they all had sufficient basic diagnostic work up, met the criteria to qualify as FUO, and most of them (49/50) had also submitted to multiple (median 3) advanced investigations. Another limitation has been the absence of an indisputable diagnostic gold standard, an obstacle common to most studies involving patients with heterogeneous nosologic background. Finally, the small sample sizes of the particular diagnostic subgroups of the present study represent an additional limitation. In order to minimize selection bias, we included consecutive adult patients with a stereotypic referral indication of FUO. Therefore, the present study aims to be representative of the cases investigated as FUO in a PET/CT academic facility of a general tertiary hospital in Greece in recent years.

Nevertheless, larger, prospective studies with more stringent referral criteria are warranted in order to further elucidate the role and timing of ^18^F-FDG-PET/CT scan in the investigation of FUO.

## 5. Conclusions

Our findings show that ^18^F-FDG-PET/CT scan is highly sensitive in either detecting causes of FUO, undetected by conventional imaging or in correctly targeting suspected lesions for successful diagnostic biopsy. The ^18^F-FDG-PET/CT contributed substantially to the diagnosis of FUO in a high percentage (70%) of our patients. Our results further support the use of ^18^F-FDG-PET/CT in the assessment of FUO.

## Figures and Tables

**Table 1:** Demographic and clinical characteristics of the study group.

Characteristic	*n* (%)	Median (IQR, Min–Max)
Number of patients	50	
Gender (male/female)	28/22 (56%/44%)	
Age (years)		59 (25, 17–85)
Concomitant Diseases/Conditions	50 (100%)	
Malignancies	8 (16%)	
Breast/ AML/H&N/URO/CR/WM	2/2/1/1/1/1	
Diabetes Mellitus	7 (14%)	
Chronic kidney desease	7 (14%)	
Cardiovascular devices	6 (12%)	
Vascular grafts/Prosthetic valves/CIED	4/1/1	
Bowel diversions	4 (8%)	
Thyroid diseases	3 (6%)	
Multinodular goiter, Hashimoto thyroiditis	2/1	
Prosthetic joints	3 (6%)	
Spinal surgery	2 (4%)	
Miscellaneous	3 (6%)	
SLE/AS/Meningioma	1/1/1	
Duration of fever (days)		40 (60, 21–365)
Common clinical/radiological findings		
Lymphadenopathy	16 (32%)	
Splenomegaly	10 (20%)	
Elevated blood inflammatory markers (ESR, CRP, PCT)	31 (62%)	
Medications	17 (34%)	
Antibiotics	13 (26%)	
Corticosteroids	1 (2%)	
Chemotherapy (last dose 4 months ago)	3 (6%)	

*n*, number of patients; IQR, interquartile range; AML, acute myeloid leukemia; H&N, head and neck cancer; URO, urothelial cancer; CR, colorectal cancer; WM, Waldenstrom macroglobulinemia; CIED, cardiac implantable electronic device; SLE, systemic lupus erythematosus; AS, ankylosing spondylitis; ESR, erythrocyte sedimentation rate; CRP, C-reactive protein; PCT, procalcitonin.

**Table 2:** Advanced investigations performed prior to PET/CT scan.

Investigation	*n* (%)
Echocardiography	20 (40%)
Computed tomography (CT)	
Thoracic CT	39 (78%)
Abdominal CT	36 (72%)
Cervical CT	8 (16%)
Cerebral CT	7 (14%)
Magnetic Resonance Imaging (MRI)	
Abdominal MRI	7 (14%)
Lumbar spine MRI	4 (8%)
Cerebral MRI	3 (6%)
Cervical spine MRI	1 (2%)
Endoscopy	
Gastroscopy	4 (8%)
Colonoscopy	4 (8%)
Bronchoscopy	3 (6%)
Nuclear medicine procedures	
^99m^Tc-MDP bone scan	2 (4%)
^99m^Tc-HMPAO-labeled leucocyte scan	1 (2%)
^99m^Tc-pertechnetate thyroid scan	1 (2%)
^99m^Tc-MAG-3 Renogram	1 (2%)
Biopsies	
Lymph node or parenchymal organ biopsy	7 (14%)
Bone marrow biopsy	9 (18%)
Temporal artery biopsy	3 (6%)

*n*, number of patients; MDP, methylene diphosphonate; HMPAO, hexamethylpropylene-amine oxime; MAG-3, mercapto-acetyl-triglycine. PET/CT, fluorodeoxyglucose positron emission tomography/computed tomography.

**Table 3:** Final diagnoses of 50 patients with fever of unknown origin (FUO).

Diagnostic Categories	*n* (%)
**Infections**	20 (40%)
Abdominal abscesses	4
Infectious cyst in polycystic renal disease	3
Pneumonia/inflammation of bronchiectasis cysts	3
Vascular graft infection	3
Tuberculous spondylitis	1
Bacterial spondylodiscitis	1
Pulmonary tuberculosis	1
CIED-associated infection	1
Infectious lymphadenopathy	1
Cryptococcosis	1
Leishmaniasis	1
**Malignancy**	8 (16%)
Non-Hodgkin’s lymphoma	5
Hodgkin’s disease	1
Lung cancer	1
Relapse of urinary tract carcinoma	1
**Non-infectious Inflammatory diseases (NIID)**	11 (22%)
Large vessel vasculitis/Takayasu’s arteritis	3
Adult-onset Still’s disease	2
Sarcoidosis	1
Polymyalgia rheumatica	1
Inflammatory bowel disease	1
Familial Mediterranean fever	1
Neo-esophagus inflammation from gastroesophageal reflux	1
Subacute thyroiditis	1
**Undiagnosed fever**	11 (22%)
Spontaneous recovery of fever	7
Recovery of fever with corticosteroids or NSAIDs	3
Recurrent fever until death	1

*n*, number of patients; CIED, cardiac implantable electronic device; NSAIDs, non-steroidal anti-inflammatory drugs.

**Table 4:** ^18^F-FDG-PET/CT scan results according to the category of final diagnosis.

Categories	TP	FP	TN	FN	*N*
Infections	19	0	0	1	20
Malignancies	8	0	0	0	8
Non-infectious inflammatory diseases	9	2	0	0	11
Undiagnosed fever	0	4	6	1	11
Total (%)	36 (72%)	6 (12%)	6 (12%)	2 (4%)	50

**Table 5:** Final diagnosis in patients with false positive or false negative ^18^F-FDG-PET/CT scans.

Gender, Age	Underlying Conditions	^18^F-FDG-PET/CT Result	Final Diagnosis	Outcome
M, 81 years	COPD, recurrent respiratory infections, prosthetic AoV	FN	No diagnosis; possible viral encephalitis	Death
M, 78 years	CIED	FN	CIED-associated infection	Fever remission after CIED removal
M, 70 years	Recurrent episodes of aspiration pneumonia; neo-esophagus due to gastrointestinal bleeding	FP: possible pulmonary aspergillosis; diffuse hypermetabolic activity along neo-esophagus	Inflammation of the neo-esophagus from gastroesophageal reflux	Spontaneous recovery
F, 57 years	THA, SLE	FP: active axillary and subclavicular lymphadenopathy (d.d. lymphoma, sarcoidosis, non-specific inflammation)	No diagnosis	Lymph node biopsy without pathological findings; spontaneous recovery
M, 49 years	Lymphadenopathy	FP: extensive (intrapelvic, inguinal, axillary), moderately active lymphadenopathy and splenic involvement; overall impression in favor of lymphoproliferative disease, (d.d. inflammatory/granulomatous etiology)	No diagnosis	Lymph node biopsy without pathological findings; recovery after antibiotics
M, 54 years	AML	FP: multiple, diffuse hypermetabolic liver lesions (d.d. infectious lesions/infiltration from hematological disease)	No diagnosis	Spontaneous recovery
M, 56 years	L3–L4 spondylodiscitis	FP: increased prostate uptake, possible prostate abscess	Adult-onset Still’s disease	Recovery with steroids
F, 63 years	Multinodular goiter, TB	FP: finding compatible with moderately active pericarditis, inactive granulomatous lung disease	No diagnosis	Recovery with steroids

M, male; F, female; FP, false positive; FN, false negative; d.d., differential diagnosis; COPD, chronic obstructive pulmonary disease; CIED, cardiac implantable electronic device; THA, total hip arthroplasty; SLE, systemic lupus erythematosus; AML, acute myeloid leukemia; TB, tuberculosis.

**Table 6:** Clinical and laboratory characteristics of patients with contributory and non-contributory scans.

Characteristic	Contributory Scans (=35) *n* (%) Mean ± SD (median)	Non-Contributory Scans (=15) *n* (%) Mean ± SD (median)	*p*-Value
Age	54.7 ± 18.6 (57.0)	62.6 ± 17.1 (63.0)	0.162
Male Female	18 (51.4) 17 (48.6)	10 (66.7) 5 (33.3)	0.609
Duration of fever (days)	72.1 ± 87.4 (30.0)	94.6 ± 84.7 (60.0)	0.080
Prior antibiotic administration	10 (28.6)	3 (20)	0.531
Lymphadenopathy	12 (34.3)	4 (26.7)	0.600
Splenomegaly	7 (20)	3 (20)	0.957
Diabetes mellitus	4 (11.4)	3 (20)	0.428
Increased CRP (mg/dL)	19 (54.3) 11.4 ± 10.0 (9.3)	3 (20.0) 7.7 ± 9.0 (3.4)	0.629 0.651
Increased ESR (mm/h)	12 (34.3) 72.7 ± 39.2 (76.5)	4 (26.7) 109.5 ± 16.7 (115.0)	0.133 0.114
Number of advanced diagnostic tests performed	3.2 ± 1.5 (3)	3.5 ± 2.0 (4)	0.463

*n*, number of patients; ESR, erythrocyte sedimentation rate; CRP, C-reactive protein.
